# Rules of co-occurring mutations characterize the antigenic evolution of human influenza A/H3N2, A/H1N1 and B viruses

**DOI:** 10.1186/s12920-016-0230-5

**Published:** 2016-12-05

**Authors:** Haifen Chen, Xinrui Zhou, Jie Zheng, Chee-Keong Kwoh

**Affiliations:** 1 School of Computer Science and Engineering, Nanyang Technological University, 50 Nanyang Avenue, 639798 Singapore, Singapore; 2 Genome Institute of Singapore, A*STAR, Biopolis, 138672 Singapore, Singapore

**Keywords:** Influenza virus, A/H3N2/H1N1 and B, Antigenic evolution, Co-occurring mutation, Influenza vaccine

## Abstract

**Background:**

The human influenza viruses undergo rapid evolution (especially in hemagglutinin (HA), a glycoprotein on the surface of the virus), which enables the virus population to constantly evade the human immune system. Therefore, the vaccine has to be updated every year to stay effective. There is a need to characterize the evolution of influenza viruses for better selection of vaccine candidates and the prediction of pandemic strains. Studies have shown that the influenza hemagglutinin evolution is driven by the simultaneous mutations at antigenic sites. Here, we analyze simultaneous or co-occurring mutations in the HA protein of human influenza A/H3N2, A/H1N1 and B viruses to predict potential mutations, characterizing the antigenic evolution.

**Methods:**

We obtain the rules of mutation co-occurrence using association rule mining after extracting HA1 sequences and detect co-mutation sites under strong selective pressure. Then we predict the potential drifts with specific mutations of the viruses based on the rules and compare the results with the “observed” mutations in different years.

**Results:**

The sites under frequent mutations are in antigenic regions (epitopes) or receptor binding sites.

**Conclusions:**

Our study demonstrates the co-occurring site mutations obtained by rule mining can capture the evolution of influenza viruses, and confirms that cooperative interactions among sites of HA1 protein drive the influenza antigenic evolution.

**Electronic supplementary material:**

The online version of this article (doi:10.1186/s12920-016-0230-5) contains supplementary material, which is available to authorized users.

## Background

Influenza has been a major and persistent threat to public health for centuries, causing millions of deaths and huge economic loss worldwide every year. Among the three types of human influenza viruses, denoted as A, B and C, influenza A viruses are the most virulent due to their high mutation rate, frequent genetic reassortment and short generation time, which have caused several pandemics in recent history [1, 2]. The pandemics include 1918 Spanish flu (A/H1N1) [3], 1957 (A/H2N2) Asia flu [4], 1968 Hongkong flu (A/H3N2) [5] and 2009 swine flu (A/H1N1) [6]. Influenza B viruses, evolving into B/Yamagata and B/Victoria lineages and frequently exchanging their segments, have been co-circulating since 2001 and cause an observably part of infections [7, 8]. Under the surveillance and monitoring by WHO (World Health Organization), influenza activity was detected to be associated with the co-circulation of influenza A/H1N1 pdm09, A/H3N2 and B viruses [9]. Therefore, to predict and prevent potential pandemics in the future, it is important to analyze and compare the evolutionary patterns of the three types of viruses.

Haemagglutinin (HA) is a surface glycoprotein of influenza virus responsible for binding specificity and initiating the viral entry. It can be cleaved into two polypeptides: HA1 and HA2 subunits, which are covalently linked by a disulfide bond [10]. HA1 contains the sialic acid receptor binding sites, and is considered as one of the main targets of immune system to detect influenza virus, as well as the primary protein component of vaccine [10–12]. Under rapid mutations (substitution rate estimated to be 5.7 × 10^− 3^per site per year [13]), the HA1 domain accumulates mutations causing viral antigenic drift and thus preclude effective vaccination with existing vaccines [14]. Identifying the evolutionary trajectories and predicting future mutations would be very helpful for recommending efficient influenza vaccines before a potential variant causes an influenza outbreak. Therefore, many studies have attempted to track and predict the antigenic evolutionary dynamics of the HA protein. Phylogenetic tree analysis is a traditional technique in this field. Studies based on phylogenetic tree analysis revealed that a single predominant trunk lineage persists through time while side branches persist for 1 ∼ 5 years before going extinct [15–17], indicating a strong selection preference in the evolutionary path. Many methods have been proposed to identify single mutation sites under positive selection and thereby understand the antigenic evolution of HA [18–20]. Statistical analysis and machine learning approaches have also been applied to reveal more information about the mutational dynamics in the viral sequences. The pioneering work by Smith et al. [21] characterized the antigenic evolution of HA1 (A/H3N2) based on the Hemagglutination-inhibition (HI) assays, and mapped the antigenic evolution (phenotype) to the phylogenetic tree based on HA1 sequences (genotype) using a maximum-likelihood (ML) approach. Smith’s method was enhanced by Bedford et al. in [22] by simultaneously characterizing antigenic and genetic evolution using a diffusion model over a shared virus phylogeny. Plotkin et al. [23] adopted a clustering technique to investigate the spatio-temporal evolution of antigenic clusters. A Bayesian approach was applied in [24] to predict the antigenic relationships of H3N2 viruses, which were used to identify the antigenic clusters and infer the dynamics of antigenic evolution. The relationship between the antigenic distances based on sequences and those calculated from HI titer data was further discussed in [25], where an online tool named “nextflu” was provided for real-time tracking. Although those studies have obtained insightful results, most of them focus on the clusters of antigenic mutations. Currently very few studies work on the interactions among site mutations in the HA proteins and their impact on the direction of antigenic evolution.

It has been observed that simultaneous multi-site mutations (or co-occurring mutations) at antigenic sites could accumulatively enhance the antigenic drift [26, 27]. Co-occurring mutations can be categorized into stochastic co-evolution, functional co-evolution and interaction evolution [28]. One site on a protein may compensate for another during evolution; thus mutations on these sites are under positive selection pressure and occur simultaneously (i.e. co-occurring mutations). The identification of co-occurring mutations can help uncover possible interactions among them and thereby improve our understanding of the mutational dynamics of proteins. Mutual information has been used to estimate the correlations between two site mutations [29, 30]. The correlation network (named site transition network or STN in [29]) based on mutual information can be used to predict the future mutations of sites in HA protein. Results in [29] showed that the STN can predict site mutations with 70% accuracy. However, mutual information is limited to pairwise relationships. How multiple sites interact with each other is yet to be discovered.

Here, we propose a method based on association rule mining [31] to identify co-occurring patterns of multiple-site mutations. Association rule mining has been shown as a promising technique in bioinformatic analysis [32, 33]. Our approach offers a flexible way to discover the interactions of multiple site mutations, not limited to pairwise interactions as in [29]. Besides, the rules of co-occurring mutations provide interpretability, making it easy for human to understand the underlying process of antigenic evolution. Furthermore, our rules can also be used to predict potential mutations in the sites of HA1.

## Results

### Rules of co-occurring mutations

First we look at all the available sequences of human influenza H1N1, H3N2 and B viruses in the Influenza Virus Resource database of NCBI [34] from 1918 to 2015. To avoid “gaps” in the data from some years, we used the sequences from 1976 to 2015 for H1N1, from 1968 to 2015 for H3N2, and from 1975 to 2015 for flu-B virus (see Methods). Using association rule mining, we discovered 24,465 rules for B virus with support and confidence larger than 5000 and 0.8 respectively. For A/H1N1 and A/H3N2 viruses, however, the support and confidence of rules are lower. Therefore, we reduced the threshold and obtained 27,704 rules for A/H1N1 with support and confidence larger than 2000 and 0.8 respectively. And for A/H3N2, there are 2266 rules with support and confidence larger than 1000 and 0.6 respectively. Details can be found in Table 1.

**Table 1:** Overview of extracted rules

Dataset	Number of Extracted Rules	Support	Confidence
A/H3N2 (1968–2015)	2266	1000	0.6
A/H1N1 (1975–2015)	27704	2000	0.8
A/H1N1 (pdm)	710	5500	0.8
A/H1N1 (aft)	181	2000	0.8
B (1976–2015)	24465	5000	0.8
B/Yamagata	1110	1500	0.8
B/Victoria	69	1500	0.8

**Fig. 1:**
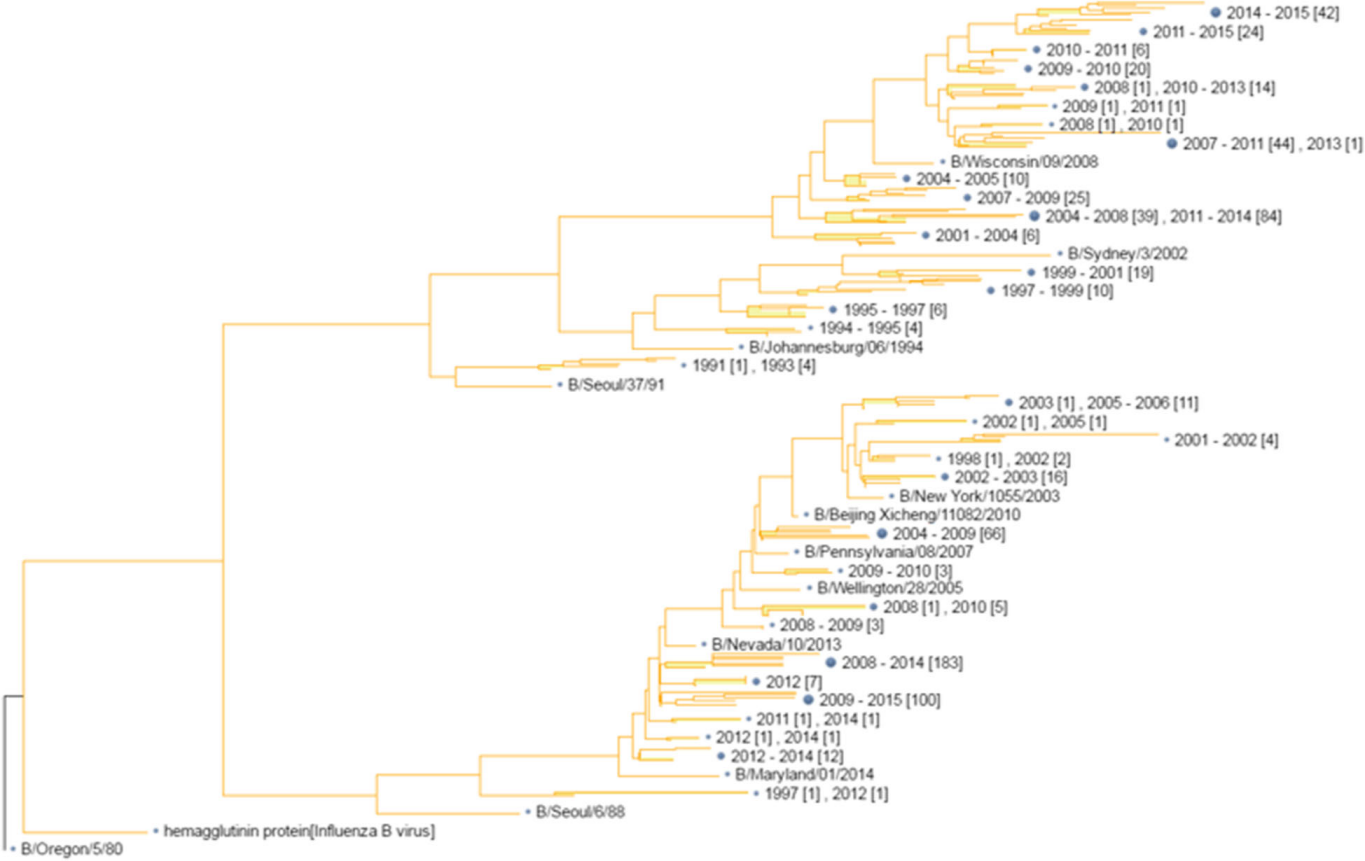
Phylogenetic tree of HA1 sequences of influenza B viruses. It’s indicated influenza virus B diverging into two lineages co-circulating around the world, named as Yamagata lineage and Victoria lineage

**Fig. 2:**
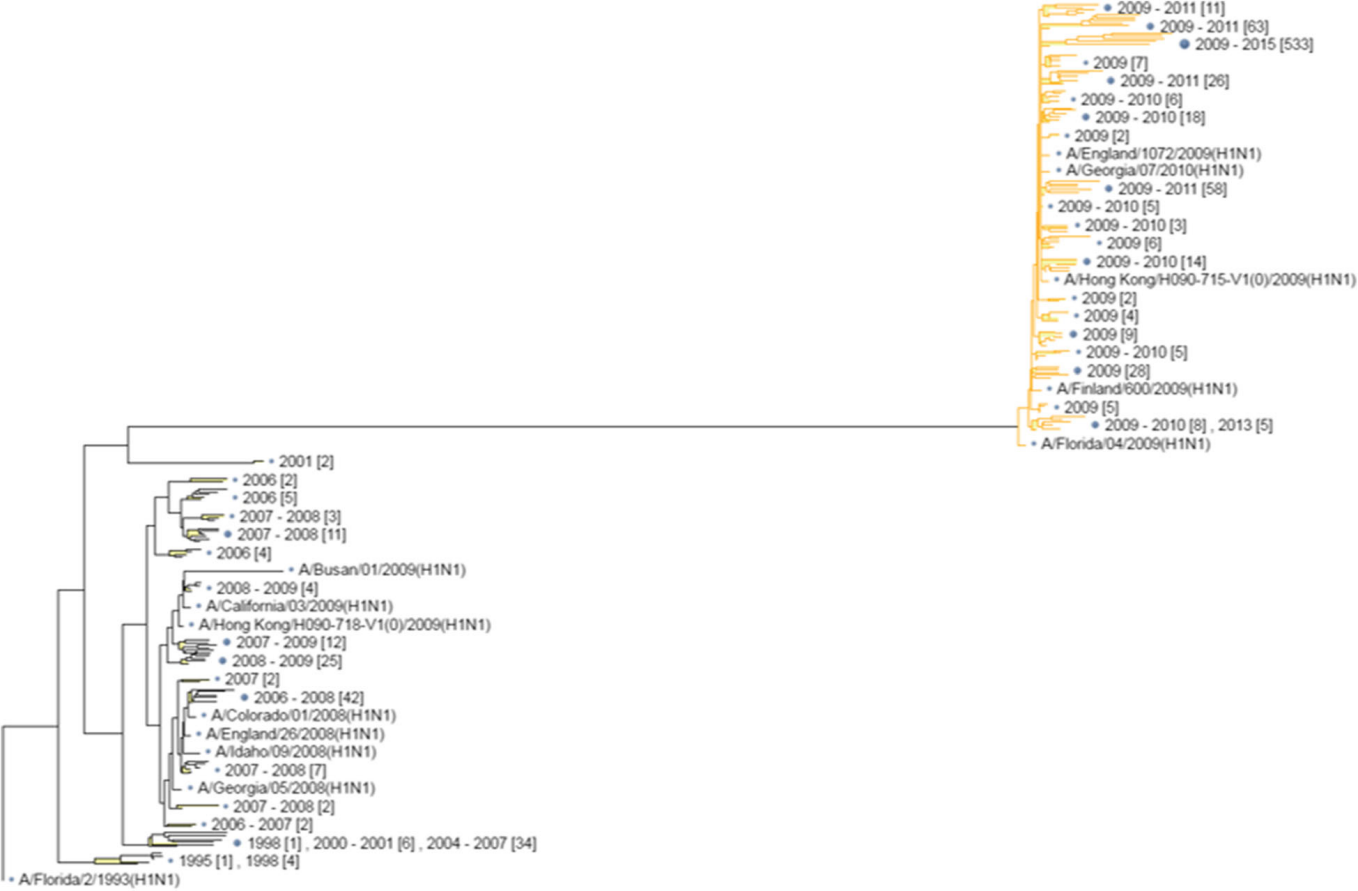
Phylogenetic tree of HA1 sequences of A/H1N1 viruses. It’s clearly divided into two clusters before and after 2009, when the world swine flu pandemic happened

**Fig. 3:**
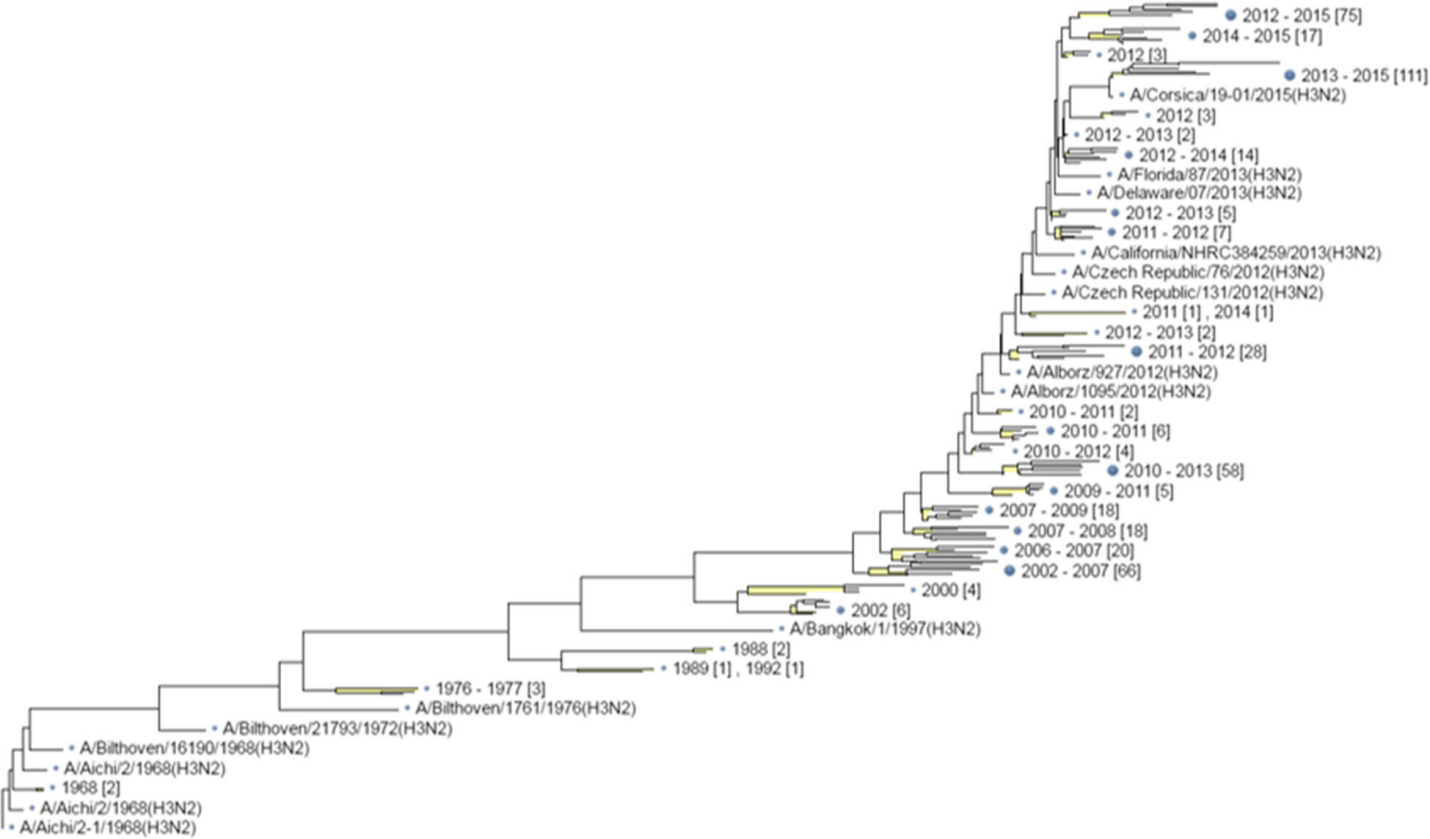
Phylogenetic tree of HA1 sequences of A/H3N2 viruses. The tree was constructed using 1000 randomly selected sequences, which showed an apparent selecting preference on the evolutionary path of A/H3N2

To compare the patterns of co-occurring mutations in different types of influenza viruses, we visualize the rules as follows. For the rules with the form X1,…,Xn= > Y , we plot a network to represent the rules, by treating X1,…,Xn as input nodes and Y as the target node. The networks of site mutations in the HA1 protein of influenza B, A/H1N1 and A/H3N2 viruses are shown in Additional file 1: Figure S1, Additional file 2: Figure S2 and Additional file 3: Figure S3. We can see that the “interactions” (i.e. the associations of items in the rules) are much denser in flu B virus than those in A/H1N1 and A/H3N2. These observations could be explained by the phylogenetic trees of the three types of influenza viruses, shown in Figs. 1, 2 and 3. Figure 1 shows that the HA1 protein sequences can be clustered into two groups (approximately corresponding to the two lineages, i.e. Victoria and Yamagata). The sequences of H1N1 are clearly divided by the year 2009, as shown in Fig. 2. For H3N2 (Fig. 3), the HA1 sequences evolve serially along the direction of years (from 1968 to 2015). Because we calculate the mutations by comparing two sequences from two adjacent years, it is not surprising that the mutations of H3N2 are less than H1N1 and B viruses. In addition, the high number of mutations found in B viruses is probably due to the fact that we did not discriminate the two lineages in this type of viruses.

Then we classified the flu-B HA1 sequences into two lineages based on their distances from two standard HA1 sequences of Victoria and Yamagata lineages. After that, we applied our approach to the sequences of the two lineages respectively. Results of rules are visualized in Additional file 4: Figure S4 and Additional file 5: Figure S5, for Yamagata and Victoria lineages respectively. We can see that, after discriminating flu-B sequences into two lineages, the numbers of mutations (within each lineage) decrease significantly. Here we set the threshold for support of rules to 1500 (versus 5000 before classifying the lineages) and obtained 1110 and 69 rules for Yamagata and Victoria lineages respectively. The results also suggest that the Yamagata lineage may mutate more quickly than the Victoria lineage.

**Fig. 4:**
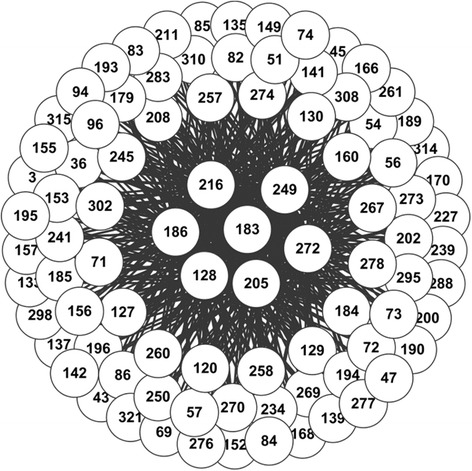
The network representing the rules of co-mutation sites in H1N1 based on sequences from 2008 to 2011

**Fig. 5:**
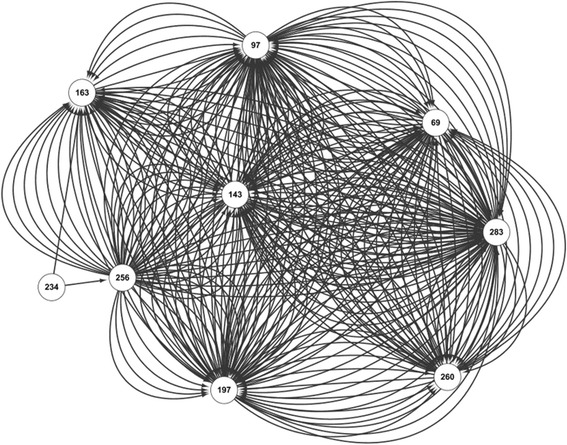
The network representing the rules of co-mutation sites in H1N1 based on sequences from 2011 to 2014

**Fig. 6:**
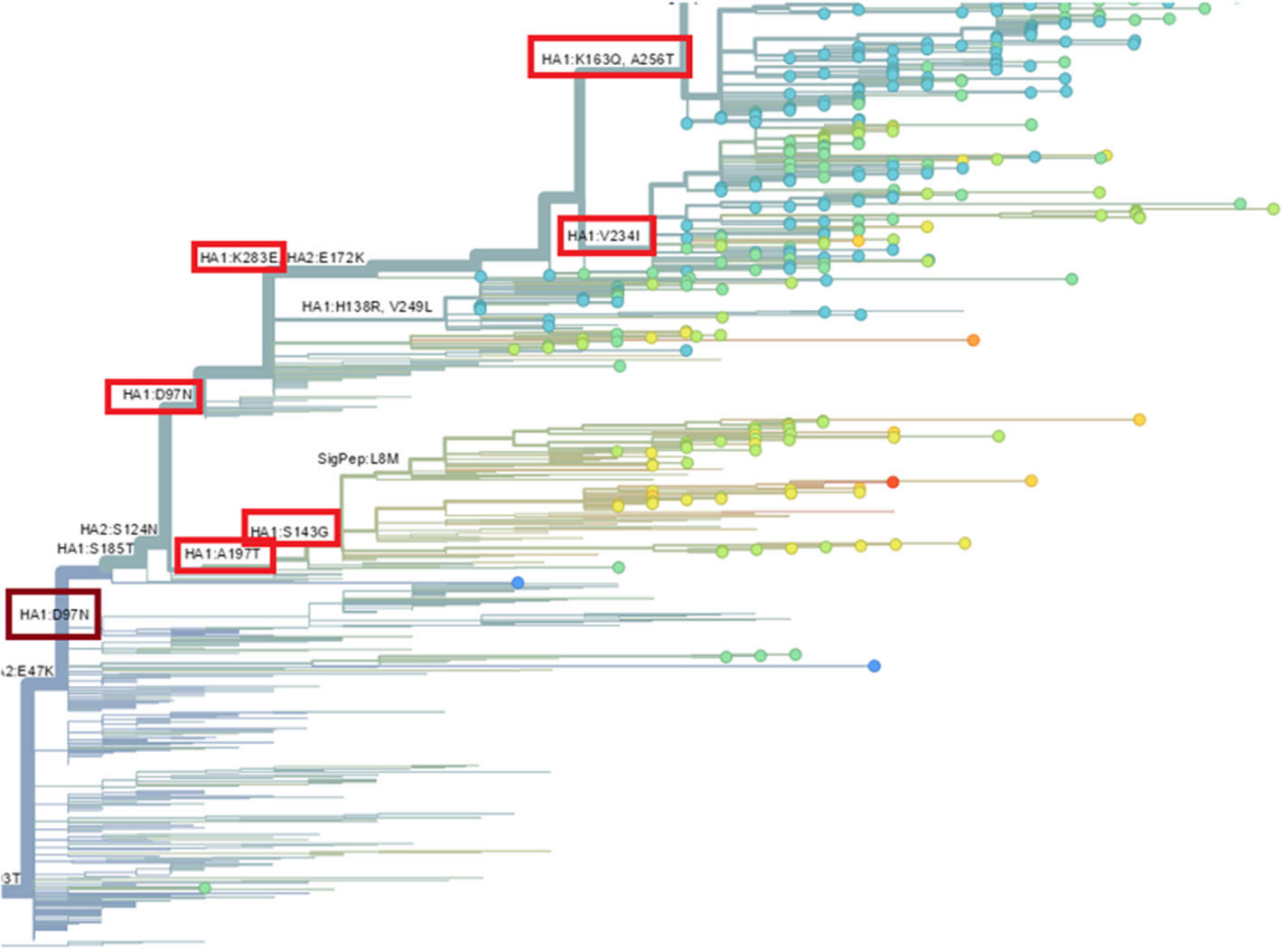
Site mutations predicted by “nextflu” [35]. The red rectangles are used to highlight the overlap with our prediction (Fig. 5)

To compare the patterns of co-occurring site mutations in A/H1N1 during and after the 2009 pandemic, we applied our method to the H1N1 sequences from 2008 to 2011 (denoted as *DATA_pdm*), and from 2011 to 2014 (denoted as *DATA_aft*) respectively. Much more mutations were obtained in *DATA_pdm* than DATA aft. Therefore, we used different thresholds for the support of the rules from the two datasets (5500 and 2000 for *DATA_pdm* and *DATA_aft* respectively). The thresholds for confidence are the same (i.e. 0.8). The rules for the two datasets are visualized as networks shown in Fig. 4 (*DATA_pdm*) and Fig. 5 (*DATA_aft*). As seen, many mutations happen in HA1 protein sequences of H1N1 during the 2009 pandemic, and most of which are “driven” by the seven site mutations: 128, 183, 186, 205, 216, 249 and 272. For *DATA_aft*, in contrast, only several sites are co-mutated frequently and they are highly inter-connected. Interestingly, these highly inter-connected sites coincide well with the mutations predicted by “nextflu” [35], as shown in Fig. 6.

The analysis of co-occurring mutation patterns in A/H3N2 is given in the section “Predictions of influenza evolution” below.

### Co-Mutation sites under strong selection pressure

**Fig. 7:**
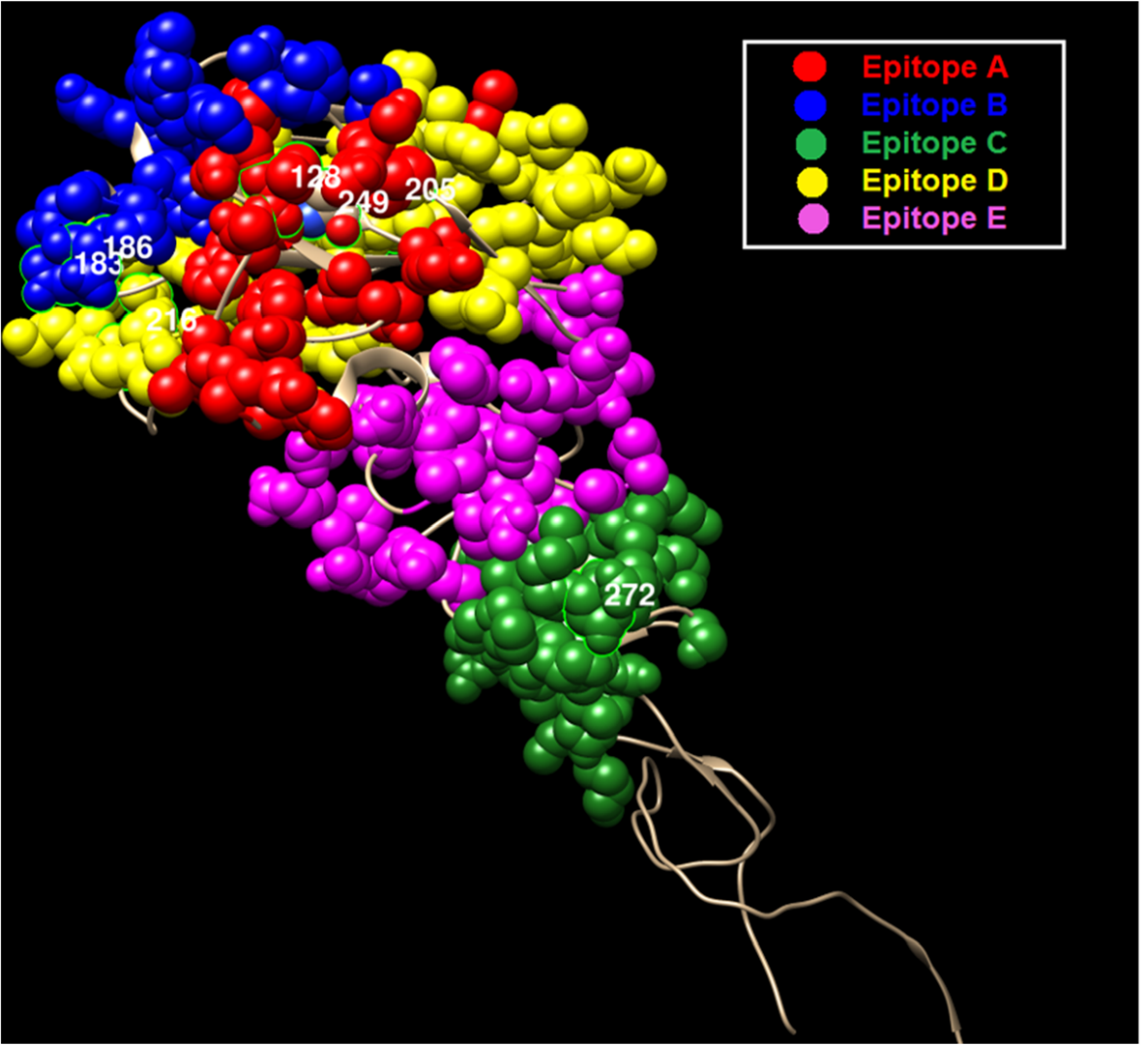
Mapping co-mutation sites on HA1 protein of H1N1 (PDB ID: 4EDB [40]). The red, blue, yellow, magenta and green colors represent the five epitope regions, the tan color denotes non-epitope region, and the co-mutation sites are circled and marked with corresponding numbers (similarly for the following two figures, i.e. Figs. 8 and 9)

**Fig. 8:**
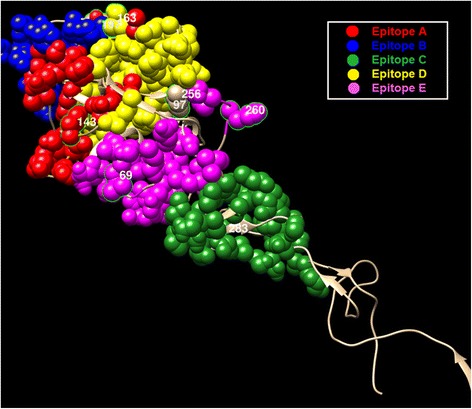
Mapping co-mutation sites on HA1 protein of H1N1 (PDB ID: 4EDB [40])

**Fig. 9:**
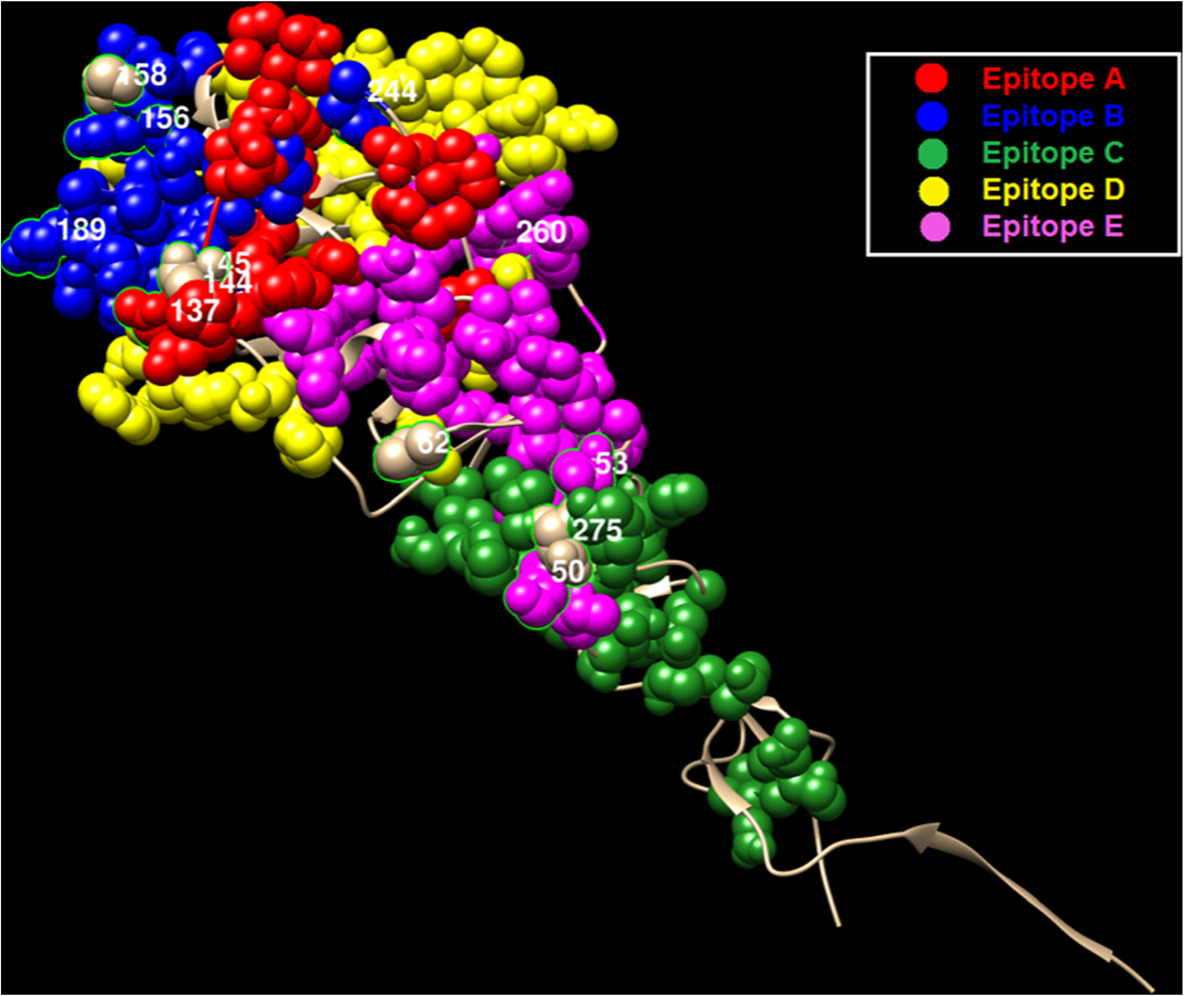
Mapping co-mutation sites on HA1 protein of H3N2 (PDB ID: 2YPG [41]). All sites are distributed on the five epitope regions, colored as red, blue, green, yellow and magenta for epitope regions A, B, C, D and E respectively

**Fig. 10:**
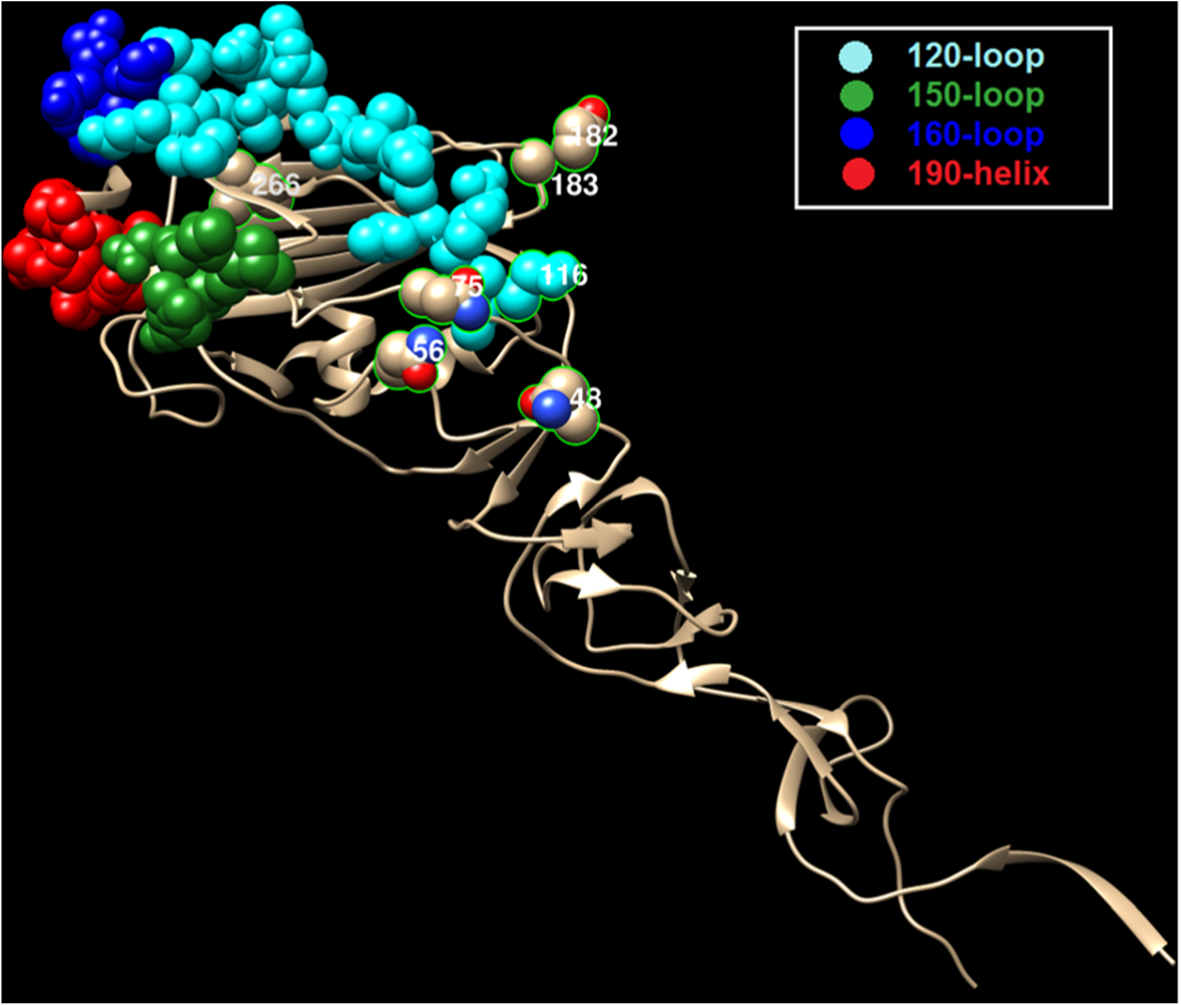
Mapping co-mutation sites detected onto HA1 protein of flu B/Yamagata (PDB ID: 4NRJ [42]). The red, blue, cyan and green colors represent 120-loop, 150-loop, 160-loop and 190-helix respectively. The four clusters have been found to cause antigenicity variation, together forming a single large antigenic site with overlapping epitopes. The co-mutation sites are circled and marked with corresponding numbers

**Fig. 11:**
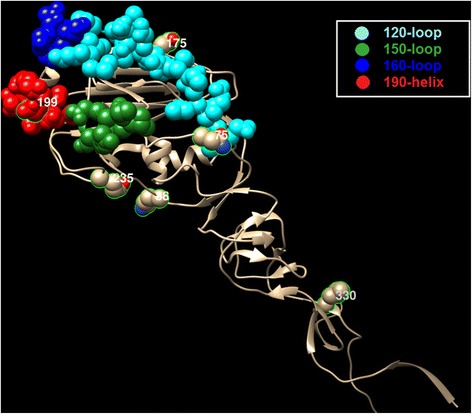
Mapping co-mutation sites detected onto HA1 protein of flu B/Victoria (PDB ID: 4NRJ [42]). The red, blue, cyan and green colors represent 120-loop, 150-loop, 160-loop and 190-helix respectively. The four clusters have been found to cause antigenicity variation, together forming a single large antigenic site with overlapping epitopes. The co-mutation sites are circled and marked with corresponding numbers

We map the co-mutation sites predicted by our method on the HA protein structures of influenza viruses. For H1N1, we have two sets of co-mutations sites, i.e. “205, 216, 183, 249, 128, 186 and 272” from Fig. 4, and “69, 97, 143, 163, 197, 256, 260 and 283” from Fig. 5. The two sets of sites are map to the HA protein structure of H1N1, as shown in Figs. 7 and 8 respectively, where the red, blue, yellow, magenta and green colors represent the five epitope regions, the tan color denotes non-epitope region, and the co-mutation sites are circled and marked with corresponding numbers. We can observe that most of the co-mutation sites fall into the epitope regions (see Table 2), indicating that these mutated sites are probably under strong selection pressure by human immune system. Similar observation has been found in the case of H3N2, shown in Fig. 9. Table 3 shows the distribution of the detected sites in H3N2 on different epitope regions. The co-mutation sites in the HA1 protein of flu-B viruses are visualized in Fig. 10 (Yamagata lineage) and Fig. 11 (Victoria lineage). There are four epitope regions in flu-B viruses, highlighted by the red, blue, cyan and green colors. The co-mutation sites are circled with green lines and marked with corresponding numbers.

**Table 2:** Sites detected co-mutated frequently with other sites

Dataset	Detected sites	Number of residues at antigenic sites/Total number of sites
A/H3N2	50, 53, 62, 137, 144, 145, 155, 156, 158, 189, 244, 260, 275	13/13
A/H1N1 (pdm)	128, 183, 186, 205, 216, 249, 272	5/7
A/H1N1 (aft)	69, 97, 143, 163, 197, 256, 260, 283	4/8
B/Yamagata	48, 56, 75, 116, 182, 183, 266	1/7
B/Victoria	75, 88, 175, 199, 330, 235	1/6

**Table 3:** Distribution of detected sites on epitope regions

Epitope A	Epitope B	Epitope C	Epitope D	Epitope E
137	155	50	244	62
144	156	53		260
145	158	275		
	189

### Predictions of influenza evolution

We predict the potential drifts with specific site mutations in HA1 protein of influenza virus A/H3N2 based on our rules (see Methods). To validate our method, we compare the predictive results from our rules with the “observed” mutations in different years (obtained from [26]). The comparison results are shown in Table 4. As seen, our method can predict the drifts with specific mutations reasonably well, especially for some years such as 1973 and 2004.

**Table 4:** The prediction results (for H3N2 in different years)

Year	Mutations										
1973	V78G	V242I	D275G	N188D	T122N	G144D	T155Y	R207K	L3F		
	✔	✔	✔	✔	✔	✔	✔	✔			
1976	F3L	D73N	T83K	T126N	I217V	I278S	Q189K	S145N			
	✔	✔	✔	✔		✔					
1978	N53D	D193N	G158E	K50R	S137Y	M260I	D2N	V244L	G146S	I62K	
							✔	✔			
2001	I226V	D133N	D124S	T121N	Y137S	R57Q	T192I	D172E	K62E	V196A	K156Q
	✔	✔	✔	✔				✔		✔	✔
2004	V144N	A131T	E83K	R50G	W222R	V202I	G225D	H155T	H75Q	Q156H	
	✔	✔	✔	✔	✔	✔	✔	✔	✔	✔	

It’s for validation of our method, where ✔ means the site mutation is predicted successfully

We also compare our results with those in [29], using the same dataset as [29] (i.e. the sequences of H3N2 from 1968 to 2002). The rules of co-mutation sites are plot as a network shown in Additional file 6: Figure S6. The following site mutations are predicted both by our method and Xia’s method (i.e. [29]): 50, 155 and 156. The mutation in site 144 is only predicted by our method. Since the “benchmark” for site mutations may not be unique, we introduce another set of “observed” mutations generated by BII-FluSurver [36]. We submitted all HA1 sequences of H3N2 in 2003 to BII-FluSurver (using default parameters) and counted the frequencies of all site mutations returned by BII-FluSurver. Totally 4543 site mutations (with duplicates) were obtained. The comparison of occurrence of predicted mutations in the BII-FluSurver results is shown in Additional file 7: Table S1, which shows that the overlap between our prediction and the BII-FluSurver results is similar to that of Xia’s prediction.

## Discussion and Conclusion

In this paper, we propose a method based on association rule mining to identify the co-occurring site mutations for human influenza A(H3N2), A(H1N1) and B Viruses. The rules of co-mutation sites characterize the antigenic evolution of influenza viruses. We show that the co-mutation sites in HA1 are all in the epitope regions, indicting strong selection pressure by human immune system in those sites. Furthermore, the rules obtained by our method can be used to predict potential mutations of influenza viruses in the future.

There are several directions to improve our study in this paper. First, we could increase the number of sampling process (i.e. N in Methods section) to increase the statistic power of association rule mining. Second, instead of randomly sampling two sequences from two adjacent years, we can select two sequences with closer phylogenetic distance to calculate the mutations. Of course, experimental data where phylogenetic relationships of sequences are known would bring better results. In addition, different weights could be assigned to different years during sampling, e.g. to do more samplings on the years with more sequences. Finally, aside from analyzing the co-occurring mutations in HA protein, we can also explore the co-evolving mutations patterns in other proteins, where mutations may compensate for each other. For example, HA and NA proteins of influenza viruses are responsible for the viral’s binding and cleavage from host cells. It would be very interesting to detect the co-occurring mutations in these two proteins, which are under selection pressure, to study their cooperative manner at the genetic level.

## Methods

### Data

All HA protein sequences of human Influenza A/H3N2, A/H1N1 and B Viruses were retrieved from the Influenza Virus Resource at NCBI [34] up to October 8, 2015. The sequences were searched from the year 1918 to the year 2015. We excluded the records without the information of year and the sequences which are shorter than the full length of HA1 (327, 312 and 345 residues for H1N1, H3N2 and B viruses, respectively).

Because there is no record in some years for a particular type of virus, we used the sequences from 1976 to 2015 for H1N1, from 1968 to 2015 for H3N2, and from 1975 to 2015 for flu-B virus, to ensure the continuity. Totally 18,450 sequences were obtained after cleaning for H1N1, 18,019 sequences for H3N2, and 6538 sequences for flu-B virus. Then we aligned these sequences using MEGA6 [37] and extracted the HA1 sequences for the three types of viruses respectively.

### Rule mining

After obtaining the HA1 sequences, we divided the sequences into different bins according to the year information. Then a technique of sampling with replacement was applied to randomly select one sequence from each bin (i.e. year). We repeated the sampling process for N times to obtain enough statistics. After that, the sequences from every two adjacent years were aligned to obtain mutations between the two sequences. The records of site mutations were treated as “transactions” in association rule mining [31], which was applied to find the rules of co-occurring site mutations. Here LCM [38] was used to carried out association rule mining.

### Mutation prediction

From the rules obtained from association rule mining, we infer which sites tend to be co-mutated during the evolution of the influenza virus. To predict potential mutations in the future, following [29], we first find out the sites under positive selection and obtain the sites co-evolving with the positive-selection sites, which would be predicted as the sites to be mutated. In [29], the positive selection site is defined as “a site that has been mutated between successive years and then remains fixed in the population for at least 1 year”. To obtain the sites under positive selection, we need to determine which sites are mutated in a particular year. Unfortunately, currently there is no standard way to obtain the yearly site mutations (i.e. the benchmark of our prediction), which make it difficult to determine the positive selection sites. Therefore, here we treat the sites occurring frequently in our rules (i.e. the sites co-mutated frequently with other sites, or with large in-degree) as the sites to be mutated.

### Visualization

Phylogenetic trees were constructed with 1000 randomly selected sequences from corresponding dataset mentioned above, using NCBI tools in “Influenza Virus Sequence Tree” [34] based on the neighbor-joining method and mPAM distance. Co-evolved sites output by our method were mapped to the virus HA protein structure retrieved from the Protein Data Bank [39–42] using Chimera (v1.11) [43]. The epitope regions of H1N1 and H3N2 are marked according to [44–47]. The epitope information of flu-B are from [48, 49].

## Additional files

Additional file 1: Figure S1. Visualization of rules for B virus (based on all HA1 sequences of flu-B from 1975 to 2015). The numbers inside the nodes denote the sites (numbering in the HA1 sequence), and the edges represent the association of the site mutations. The same applies to the rest figures of “Visualization of rules”. (PDF 673 kb)
Additional file 2: Figure S2. Visualization of rules for A/H1N1 virus (based on all HA1 sequences of H1N1 from 1976 to 2015). (PDF 511 kb)
Additional file 3: Figure S3. Visualization of rules for A/H3N2 virus (based on all HA1 sequences of H3N2 from 1968 to 2015). (PDF 286 kb)
Additional file 4: Figure S4. The network representing the rules of co-mutation sites in B viruses (Yamagata lineage). (PDF 314 kb)
Additional file 5: Figure S5. The network representing the rules of co-mutation sites in B viruses (Victoria lineage). (PDF 230 kb)
Additional file 6: Figure S6. Network of co-mutation sites based on H3N2 sequences from 1968 to 2002 (same dataset as in Xia et al.). (PDF 511 kb)
Additional file 7: Table S1. Comparison of site mutations prediction (for H3N2 in the year 2003). (PDF 224 kb)
