# Supplementary material for: Rules of co-occurring mutations characterize the antigenic evolution of human influenza A/H3N2, A/H1N1 and B viruses
Source: BMC Med Genomics. 2016 Dec 5;9(Suppl 3):69. doi: 10.1186/s12920-016-0230-5 (PMC5260787; doi:10.1186/s12920-016-0230-5)
Supplement: Additional file 1: Figure S1. — Visualization of rules for B virus (based on all HA1 sequences of flu-B from 1975 to 2015). The numbers inside the nodes denote the sites (numbering in the HA1 sequence), and the edges represent the association of the site mutations. The same applies to the rest figures of “Visualization of rules”. (PDF 673 kb) [file 12920_2016_230_MOESM1_ESM.pdf]

Additional File 1. Figure S1. Visualization of rules for B virus (based on all HA1 sequences of flu-B from 1975 to 2015).

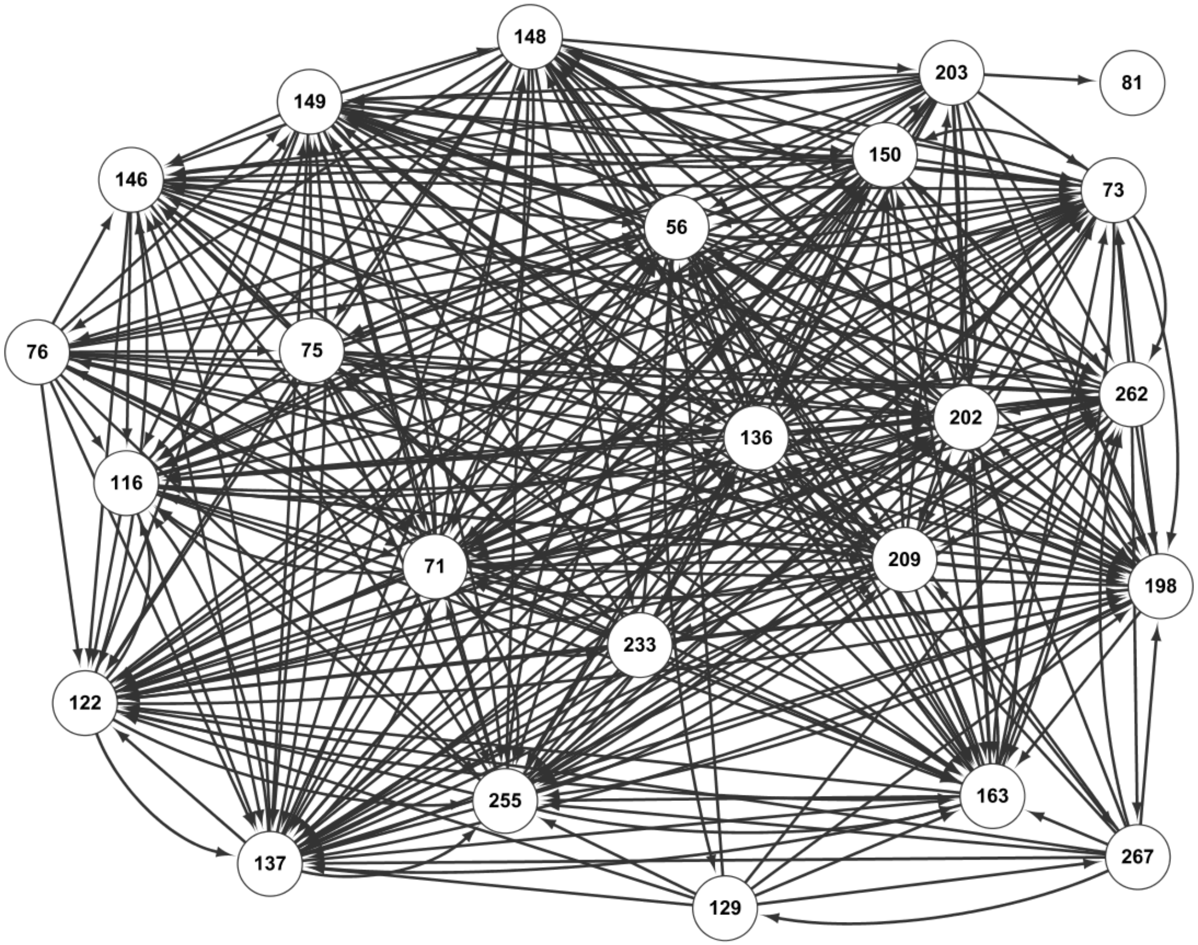

The numbers inside the nodes denote the sites (numbering in the HA1 sequence), and the edges represent the association of the site mutations. The same applies to the rest figures of "Visualization of rules".
