# Supplementary material for: Rules of co-occurring mutations characterize the antigenic evolution of human influenza A/H3N2, A/H1N1 and B viruses
Source: BMC Med Genomics. 2016 Dec 5;9(Suppl 3):69. doi: 10.1186/s12920-016-0230-5 (PMC5260787; doi:10.1186/s12920-016-0230-5)

Additional File 2. Figure S2. Visualization of rules for A/H1N1 virus (based on all HA1 sequences of H1N1 from 1976 to 2015)

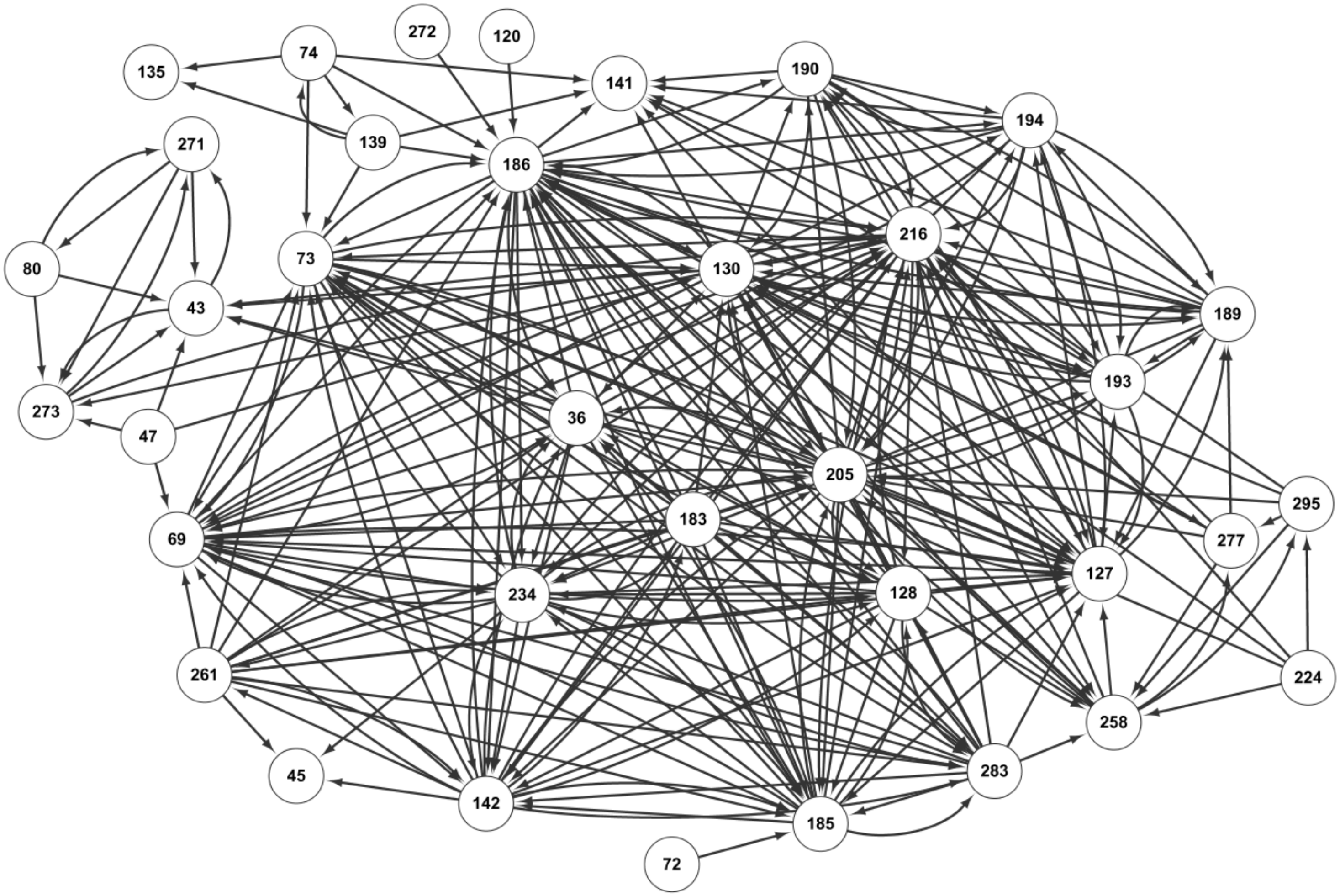

Supplement: Additional file 2: Figure S2. — Visualization of rules for A/H1N1 virus (based on all HA1 sequences of H1N1 from 1976 to 2015). (PDF 511 kb) [file 12920_2016_230_MOESM2_ESM.pdf]
