# Supplementary material for: Rules of co-occurring mutations characterize the antigenic evolution of human influenza A/H3N2, A/H1N1 and B viruses
Source: BMC Med Genomics. 2016 Dec 5;9(Suppl 3):69. doi: 10.1186/s12920-016-0230-5 (PMC5260787; doi:10.1186/s12920-016-0230-5)

Additional File 3. Figure S3. Visualization of rules for A/H3N2 virus (based on all HA1 sequences of H3N2 from 1968 to 2015).

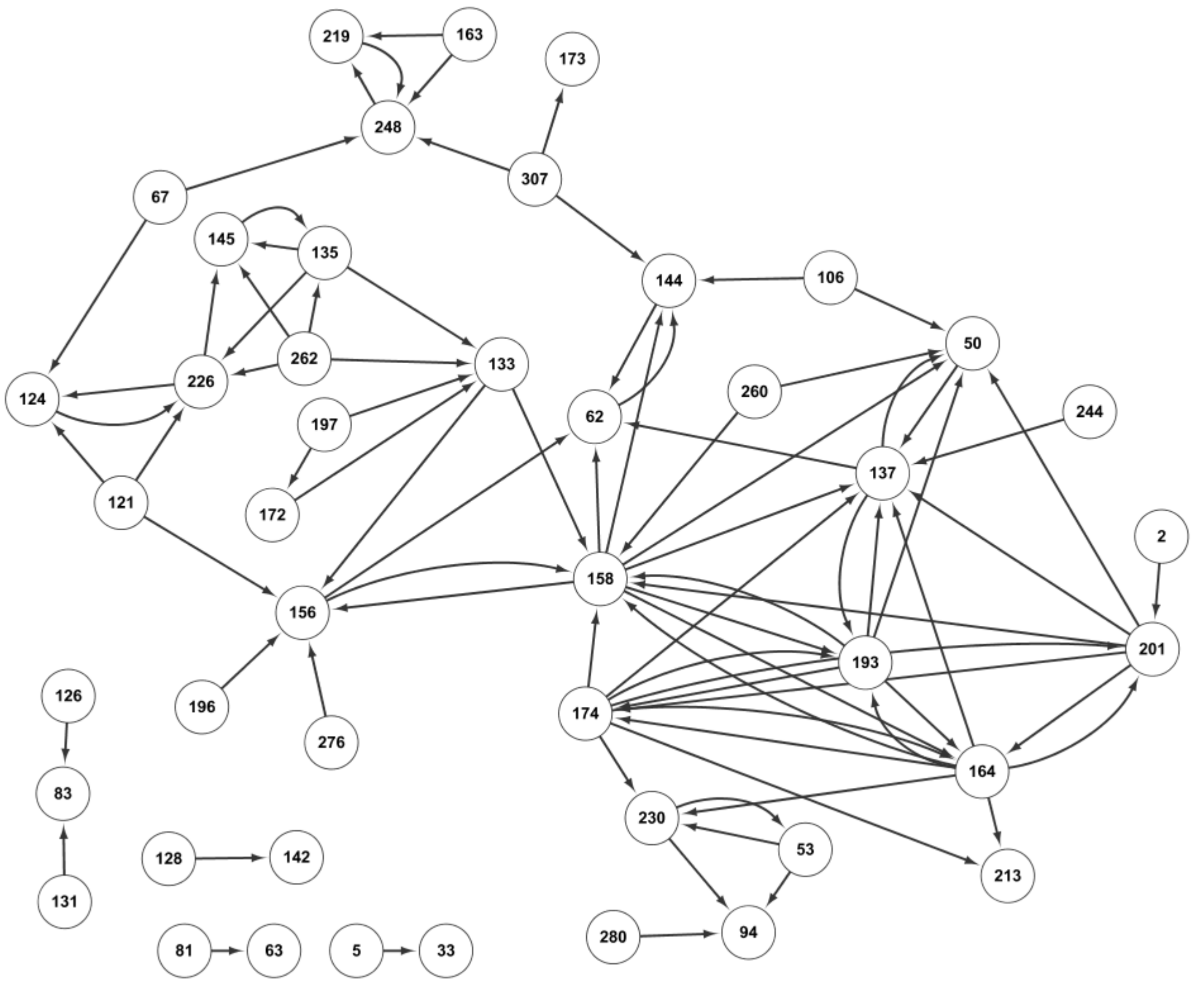

Supplement: Additional file 3: Figure S3. — Visualization of rules for A/H3N2 virus (based on all HA1 sequences of H3N2 from 1968 to 2015). (PDF 286 kb) [file 12920_2016_230_MOESM3_ESM.pdf]
