# Supplementary material for: Rules of co-occurring mutations characterize the antigenic evolution of human influenza A/H3N2, A/H1N1 and B viruses
Source: BMC Med Genomics. 2016 Dec 5;9(Suppl 3):69. doi: 10.1186/s12920-016-0230-5 (PMC5260787; doi:10.1186/s12920-016-0230-5)

Additional File 4. Figure S4. The network representing the rules of co-mutation sites in B viruses (Yamagata lineage).

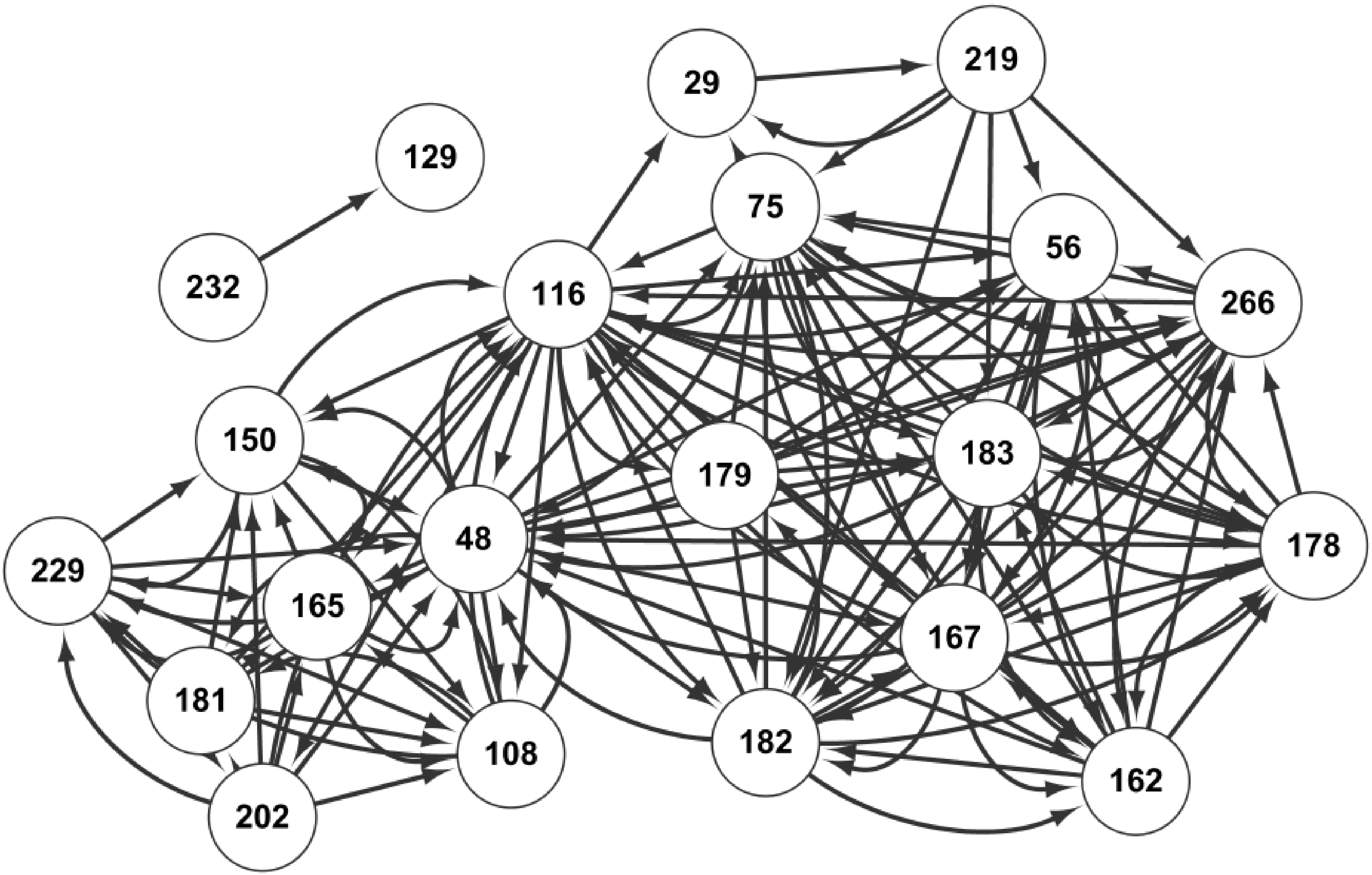

Supplement: Additional file 4: Figure S4. — The network representing the rules of co-mutation sites in B viruses (Yamagata lineage). (PDF 314 kb) [file 12920_2016_230_MOESM4_ESM.pdf]
