# Supplementary material for: Rules of co-occurring mutations characterize the antigenic evolution of human influenza A/H3N2, A/H1N1 and B viruses
Source: BMC Med Genomics. 2016 Dec 5;9(Suppl 3):69. doi: 10.1186/s12920-016-0230-5 (PMC5260787; doi:10.1186/s12920-016-0230-5)

Additional File 6. Figure S6. Network of co-mutation sites based on H3N2 sequences from 1968 to 2002 (same dataset as in Xia et al.)

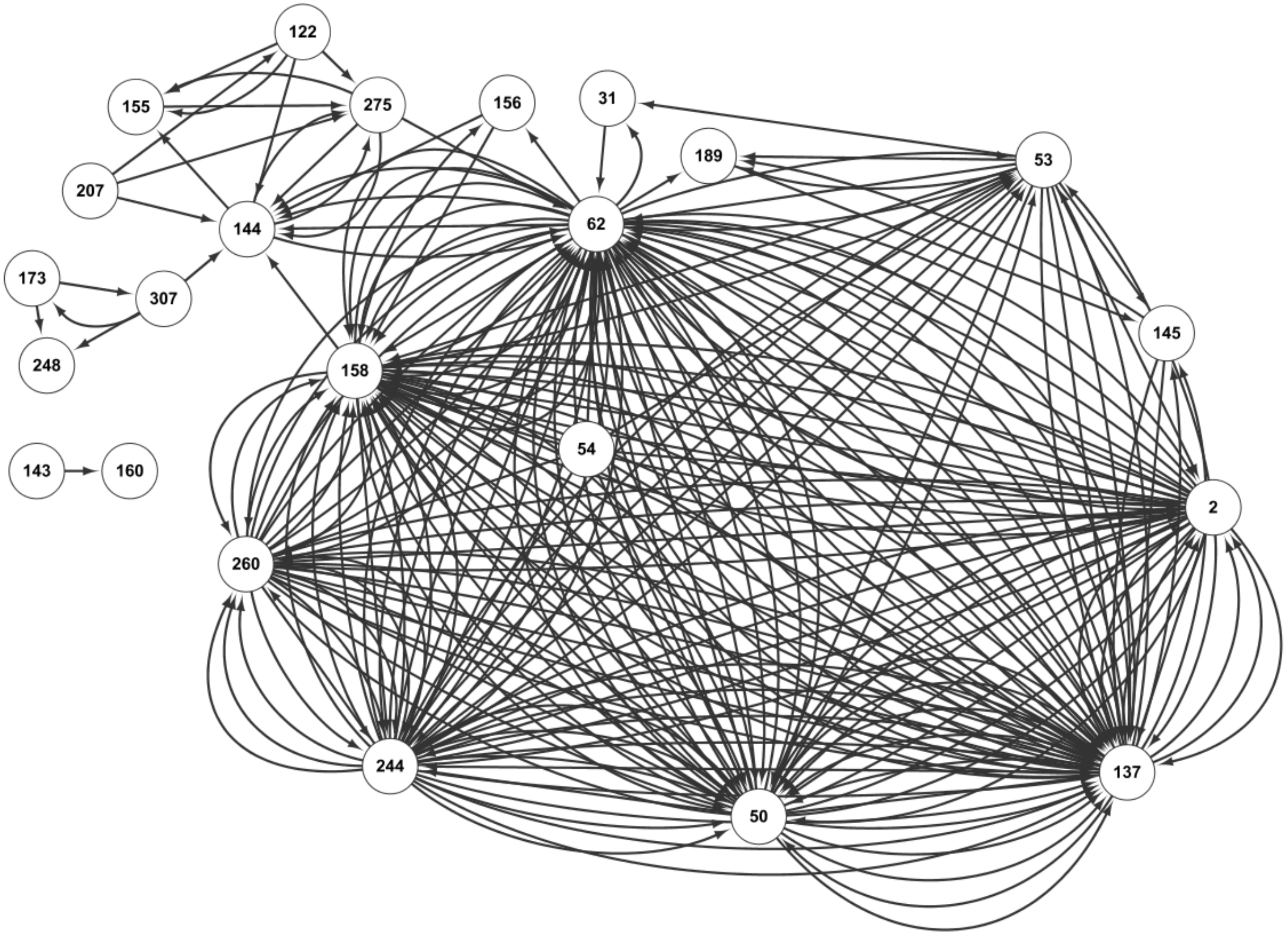

Supplement: Additional file 6: Figure S6. — Network of co-mutation sites based on H3N2 sequences from 1968 to 2002 (same dataset as in Xia et al.). (PDF 511 kb) [file 12920_2016_230_MOESM6_ESM.pdf]
