# Supplementary material for: Rules of co-occurring mutations characterize the antigenic evolution of human influenza A/H3N2, A/H1N1 and B viruses
Source: BMC Med Genomics. 2016 Dec 5;9(Suppl 3):69. doi: 10.1186/s12920-016-0230-5 (PMC5260787; doi:10.1186/s12920-016-0230-5)
Supplement: Additional file 7: Table S1. — Comparison of site mutations prediction (for H3N2 in the year 2003). (PDF 224 kb) [file 12920_2016_230_MOESM7_ESM.pdf]

**Additional File 7. Table S1. Comparison of site mutations prediction (for H3N2 in the year 2003).**

| | | | | | | | | | | | |
|---|---|---|---|---|---|---|---|---|---|---|---|
| Observed in [28] | 50 | 75 | 83 | 131 | 144 | 155 | 156 | 186 | 202 | 222 | 225 |
| Occurrence in FluSurver Results | 4 | 1 | 0 | 0 | 22 | 0 | 374 | 0 | 12 | 1 | 3 |
| Predicted by [28] | 50 | 75 | 131 | 155 | 156 | 202 | 222 | | | | |
| Occurrence in FluSurver results | 4 | 1 | 0 | 0 | 374 | 12 | 1 | | | | |
| Predicted by our rules | 50 | 53 | 62 | 137 | 144 | 155 | 156 | 158 | 244 | 260 | 275 |
| Occurrence in FluSurver results | 4 | 0 | 1 | 7 | 22 | 0 | 374 | 11 | 6 | 0 | 1 |

The table compares the site mutations prediction of our method, the approach in Xia et al [29] and occurrence of predicted mutations in the BII-FluSurver [44]. The overlap between our prediction and the BII-FluSurver results is similar to that of Xia's prediction.
